# Characteristics of drug-related problems and pharmacist’s interventions in hospitalized patients in Thailand: a prospective observational study

**DOI:** 10.1038/s41598-022-21515-7

**Published:** 2022-10-12

**Authors:** Kulchalee Deawjaroen, Jutatip Sillabutra, Nalinee Poolsup, Derek Stewart, Naeti Suksomboon

**Affiliations:** 1 Department of Pharmacy, Faculty of Pharmacy, Mahidol University, Bangkok, Thailand; 2 Faculty of Public Health, Mahidol University, Bangkok, Thailand; 3 Samrejvittaya School, Aranyaprathet, Sakaeo Thailand; 4 College of Pharmacy, QU Health, Qatar University, Doha, Qatar

**Keywords:** Diseases, Health care, Medical research, Risk factors

## Abstract

Drug-related problems (DRPs) are a major health concern. A better understanding of the characteristics of DRPs throughout the hospital stay may help to tailor pharmaceutical care services (PCS). This study aims to describe the characteristics of DRPs and to compare DRP pattern in different stages of hospital stay. DRPs were identified by clinical pharmacists as part of their routine services. Pharmacist assessed causality, severity and preventability of DRP. A total of 316 preventable DRPs occurred in 257 patients with the median of 1 (rang 1–3) DRPs per patient. 46.8% of DRPs occurred at discharge than at other stages. The most frequent cause of DRP was no drug treatment in spite of existing indication, accounting for 32.3% of all DRPs. No drug treatment with existing indication was detected frequently at discharge (56.1%) compared with other stages (p-value < 0.001). The common intervention to physician was starting a drug (34.0%) and the acceptance rate was 95.8%. DRPs in hospitalized patients occur at any stage of the hospital stay. Systematic identification of DRP characteristics enables pharmacists to tailor optimal type of PCS required and hence improve patient safety.

## Introduction

Drug-related problems (DRPs), defined as “events involving drug therapy that actually or potentially interfaces with the patient experiencing an optimum outcome of medical care”^1^, are an important problem in healthcare systems worldwide, as they are associated with patient harm and increased economic burden^2–5^. Consequently, the third World Health Organization Global Patient Safety Challenge was launched to address this major issue. It aimed to reduce severity and avoidable medication-related harm by 50% within 5 years^3^. Hospitalized patients are vulnerable to the occurrence of DRPs due to their acute condition leading to admission, and frequent changes in drug regimen (e.g., initiation or discontinuation, dose adjustment)^6–8^. Globally, the incidence of DRPs among hospitalized patients has been reported in studies as ranging from 15.5 to 81.0%, with half being potentially preventable^6,7,9–12^.

To prevent or minimize the DRP, having an effective strategy for identification is essential. A pharmaceutical care service (PCS) is a key strategy to identify and resolve DRPs, including medication reconciliation, medication review and the integration of pharmacists into the health care teams^13,14^. Studies in several countries have shown the integration of PCS by clinical pharmacist involved in patient care resulted in a reduction of DRPs and improving patient safety^14,15^. However, it is challenging to consistently and continuously deliver comprehensive PCS to all patients due to a shortage of trained staff together with an increasing number of patients admitted^16–18^. These findings highlight the need for tailored PCS.

A better understanding of the characteristics of DRPs throughout hospital stay may provide increased insight into the types of PCS required. Studies to investigate DRPs among hospitalized patients have been conducted^6,9,11,12^, however these studies focused on the incidence and characteristics of DRPs only at admission or transition care or at discharge. There were a few studies addressing the stages of hospital stay in the identification and resolution of DRPs. Recently, Geeson et al.^19^ conducted an analysis of the categories of clinically relevant medication-related problems (MRPs) both overall and by stage of hospital stay. Their results showed that clinically relevant MRPs were more frequently identified during/before the first ward-based pharmacy review of patients (73.9% of all MRPs). Currently, no study focuses on characteristics of DRPs along with stages of hospital stay in Thailand. Therefore, these data may inform the main problems and the design of PCS. The aims of the study were to describe the characteristics of DRPs in hospitalized patients at a tertiary hospital in Thailand, and to compare DRP pattern in different stages of hospital stay. In addition, this study examined the type and acceptance of pharmacists’ interventions.

## Methods

### Study design and participants

This study was a prospective observational study conducted from January to July 2020 at Lampang hospital, a 800-bed tertiary hospital in Northern Thailand. A consecutive sampling technique was employed to select patients based on the study period. Adult patients aged ≥ 18 years admitted to general medical wards and hospitalized for more than 24 h were included. Patients were excluded if their prescribing records were not reviewed by clinical pharmacists during their admissions or medical records were unavailable. The sample size was calculated. At least 246 patients were recruited assuming a DRP rate of 0.2 and a confidence level of 0.95 with margin of error at 5%^9,10,20^. All eligible patients were taking account for analysis.

The study was approved by the Institutional Review Board of the Faculty of Dentistry/Faculty of Pharmacy, Mahidol University, Thailand (No.2019/067.0110) and by the Research Committee at Lampang Hospital (No. 135/63). These Ethics Committee waived the need to obtain informed consent from patients since the research obtained only secondary data. Secondary data was collected from medical records and electronic hospital databases, as part of standard practice for patient care. All study procedures and processes were conducted in accordance with the Declaration of Helsinki and with the ethical standards of the institutional and/or research committee.

### Data collection

Clinical pharmacists performed daily medication reviews and reconciliation to identify DRPs on the basis of pharmaceutical care. An intervention was given to physician in order to resolving DRPs such as drug selection, dosage regimens, and possible drug monitoring needs. Physician making the final decision. Initial DRP screening and suspected DRPs were identified by clinical pharmacists at the study site. Principal researcher (KD) and a senior clinical pharmacist at study site independently reviewed DRP. Data were collected from admission until discharge. Each identified DRPs were recorded into a data collection form. Problems and causes of DRPs were assessed using the Pharmaceutical Care Network Europe (PCNE) classification V8.02^1^. In addition, severity and preventability of these DRPs were assessed using the National Coordinating Council for Medication Error Reporting and Prevention (NCC MERP)^21^ and the Hepler and Strand criteria^13^, respectively. Any discrepancies in assessments were resolved by consensus (NS and NP; senior clinical pharmacists). The interventions and acceptance of these recommendation were documented.

Patient demographic, clinical and laboratory data as well as regimen were collected by the principal researcher (KD) through medical chart and electronic hospital database. The following data were recorded: age, gender, body weight, history of drug allergies, relevant medical and medication history, drugs used during the hospital stay and at discharge, routine laboratory reports and the diagnosed diseases which are important for identification of DRP. Drugs involved in DRPs were recorded and coded in accordance with the Anatomical Therapeutic Classification (ATC) classification system^22^.

### Data analysis

The preventable DRPs according to the Hepler and Strand criteria were included for descriptive analysis.

Descriptive statistics were performed to describe demographic, clinical data, and DRP related data. The continuous data were test normality by histogram and skewness values. The data such as age, number of comorbidities and number of drugs were approximately normal distribution (skewness values between − 1 and + 1) (Supplementary information S1). Results were expressed as mean along with standard deviation (SD). Categorical data were expressed as frequency and percentage. Chi-square test or Fisher’s exact test was performed to test the differences in proportions of each DRP subcategory among the stages of hospital stay. P-value of < 0.05 was considered statistically significant. Clopper–Pearson interval was also used to determine 95% confidence interval (CI) for proportions regarding to the stages of hospital stay. All data were analyzed using SPSS statistics version 18.0.

## Results

**Figure 1:**
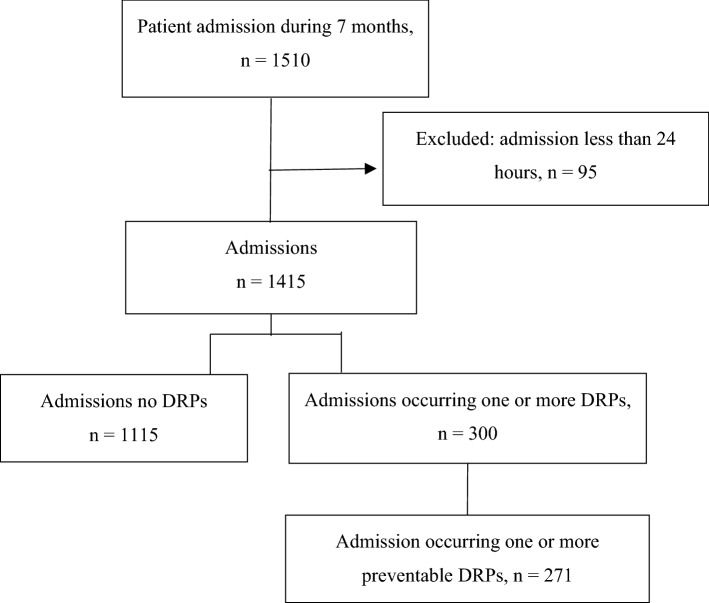
Participant flow diagram.

During the seven-month study period, 1510 patient admissions were consecutively included into the study. Of these admissions, 95 were excluded due to their admission less than 24 h and 1415 were remaining. Among 1415 admissions, 271 admissions (257 patients) were identified as having at least one preventable DRP (Fig. 1). Of the 257 patients occurring DRP, 213 (82.9%) had one, 40 (15.6%) had two, and 4 (1.5%) had three DRPs. Mean age of the patients was 67.30 (15.69) years, and 59.5% were males. The majority of patients (92.6%, n = 238) had comorbidities and mean number of baseline comorbidities was 2.68 (1.50). The most common comorbidities were hypertension (59.9%), dyslipidemia (44.0%) and diabetes (30.0%). The mean number of drugs used was 5.72 (3.76). Approximately two-third of patients (n = 165) took 5 or more drugs per day.

### Characteristics of drug-related problems

A total of 316 preventable DRPs were detected during 271 admissions (257 patients), giving a median of 1 (range 1–3) preventable DRPs per patient. Considering the severity of the 316 preventable DRPs, 145 (45.9%) were rated as NCC MERP category B (an DRP occurred but the DRP did not reach the patients), 131 (41.5%) were category C (a DRP occurred that reached the patient but did not cause patient harm) and 40 (12.7%) were category D (a DRP occurred that reached the patient and required monitoring to confirm that it resulted in no harm to the patient and/or required intervention to preclude harm). Approximately, half of the DRPs (n = 148) were detected at the hospital discharge.

According to the PCNE V8.02 classification (Table 1), the principal problems of the DRPs were treatment effectiveness and treatment safety. The most common subcategories were adverse drug events (n = 116, 36.7%), followed by untreated symptoms or indication (n = 97, 30.7%) and effect of drug treatment not optimal (n = 55, 17.4%). When expressed by the stages of hospital stay, there were significantly difference in four subcategories of DRPs, including effect of drug treatment not optimal (p = 0.001), untreated symptoms/indication (p < 0.001), adverse drug events (p < 0.001), and unnecessary drug-treatment (p < 0.001). Detail of 95% CI was reported in Supplementary information S2. Untreated symptoms or indication was often detected at discharge (53.4%), compared with at admission and during the hospital stay (11.7% and 9.9%, respectively). While the remaining categories were identified at admission and during the hospital stay.

**Table 1:** Problems of drug-related problems identified among three stages of hospital stay.

Category/subcategory of DRP problems according to PCNE v 8.02 classification	Number of DRPs divided by stage of DRPs identified, n (% of each stage)			Total, n = 316 n (% of total)	p-value (test for difference among stages)
	At admission, n = 77	During the hospital stay, n = 91	At discharge, n = 148		
**Treatment effectiveness**					
No effect of drug treatment	1 (1.3)	2 (2.2)	3 (2.0)	6 (1.9)	1.000^b^
Effect of drug treatment not optimal	24 (31.2)	10 (11.0)	21 (14.2)	55 (17.4)	0.001^a^
Untreated symptoms or indication	9 (11.7)	9 (9.9)	79 (53.4)	97 (30.7)	< 0.001^a^
**Treatment safety**					
Adverse drug event (possibly) occurring	38 (49.4)	43 (47.3)	35 (23.6)	116 (36.7)	< 0.001^a^
**Other problems**					
Unnecessary drug-treatment	5 (6.5)	27 (29.7)	10 (6.8)	42 (13.3)	< 0.001^a^

*DRP* drug-related problem, *PCNE* Pharmaceutical Care Network Europe.

^a^p-value of Chi-square for comparing three groups, statistical significance 0.05.

^b^p-value of Fisher exact test for comparing two groups: one group was DRPs occurred during admission (combination of at admission and during the hospital stay) and another was the DRPs at discharge, statistical significance 0.05.

Overall, there were five categories of the DRP identified in this study, as summarized in Table 2.

**Table 2:** Causes of drug-related problems identified among three stages of hospital stay.

Category/subcategory of DRP causes according to PCNE v 8.02 classification	Number of DRPs divided by stage of DRPs identified, n (% of each stage)			Total, n = 316 n (% of total)	p-value (test for difference among stages)
	At admission, n = 77	During the hospital stay, n = 91	At discharge, n = 148		
**Drug selection**					
Inappropriate drug according to guidelines/formulary	4 (5.2)	8 (8.8)	5 (3.4)	17 (5.4)	0.211^b^
Inappropriate drug (within guidelines but otherwise contraindication)	9 (11.7)	6 (6.6)	4 (2.7)	19 (6.0)	0.026^a^
No indication	2 (2.6)	1 (1.1)	3 (2.0)	6 (1.9)	> 1.000^b^
Inappropriate combination of drugs or drugs and herbal medication	0	1 (1.1)	1 (0.7)	2 (0.6)	> 1.000^b^
Inappropriate duplication of therapeutic group or active ingredient	3 (3.9)	16 (17.6)	5 (3.4)	24 (7.6)	< 0.001^a^
No drug treatment in spite of existing indication	11 (14.3)	8 (8.8)	83 (56.1)	102 (32.3)	< 0.001^a^
Too many drugs prescribed for indication	0	8 (8.8)	2 (1.4)	10 (3.2)	0.111^b^
**Drug form**					
Inappropriate dosage form	2 (2.6)	3 (3.3)	4 (2.7)	9 (2.9)	1.000^b^
**Dose selection**					
Dose too low	13 (16.9)	5 (5.5)	8 (5.4)	26 (8.2)	0.006^a^
Dose too high	22 (28.6)	28 (30.8)	22 (14.9)	72 (22.8)	0.007^a^
Dosage regimen not frequent enough	2 (2.6)	0	0	2 (0.6)	0.500^b^
Dose timing instructions wrong, unclear or missing	8 (10.4)	2 (2.2)	6 (4.1)	16 (5.1)	0.609^b^
**Treatment duration**					
Duration of treatment too short	0	0	2 (1.4)	2 (0.6)	0.219^b^
Duration of treatment too long	0	4 (4.4)	0	4 (1.3)	0.126^b^
**Others**					
Inappropriate outcome monitoring	0	1 (1.1)	0	1 (0.3)	1.000^b^
Drug order incorrect or incomplete	1 (1.3)	0	3 (2.0)	4 (1.3)	0.344^b^

*DRP* drug-related problem, *PCNE* Pharmaceutical Care Network Europe.

^a^p-value of Chi-square for comparing three groups, statistical significance 0.05.

^b^p-value of Fisher exact test for comparing two groups: one group was DRPs occurred during admission (combination of at admission and during the hospital stay) and another was the DRPs at discharge, statistical significance 0.05.

The most frequent DRP were no drug treatment in spite of existing indication (32.3%), dose too high (22.8%), and dose too low (8.2%). Regarding the stages of DRP identified, there were five subcategories of the DRP causes having significant differences among three stages of hospital stay, including inappropriate drug (inline with the guidelines but otherwise contraindication) (p = 0.026), inappropriate duplication of therapeutic group (p < 0.001), no drug treatment despite existing indication (p < 0.001), dose too low (p = 0.006), and dose too high (p = 0.007). The detail of 95% CI was reported in Supplementary information S2. For example, the highest percentage of the subcategory “no drug treatment in spite of existing indication” was detected at discharge (56.1%) compared with at admission (14.3%) and during the hospital stay (8.8%).

However, the observed frequency of some subcategories were too small to show the differences among three stages. Consequently, Fisher’s exact test was used to compare the difference in each subcategory between the two groups; one group was DRPs occurred during admission (combination of at admission and during the hospital stay) and another was the DRPs at discharge. There was no significant difference of each DRP across stages when using Fisher’s exact test.

### Drug involved in drug-related problems

A total of 385 drugs were involved in the DRPs. The top five ATC therapeutic classes were cardiovascular system (27.5%), anti-infectives for systematic use (27.0%), alimentary tract and metabolism (15.8%), blood and blood forming organs (11.2%), and respiratory system (6.5%). Individual drugs that most encountered in DRPs were atorvastatin (n = 26), omeprazole (n = 25), warfarin (n = 17), piperacillin and tazobactam (n = 11), and amlodipine (n = 10) (Table3).

**Table 3:** Summary of ATC classes and drug involved in drug-related problems.

ATC class	Total, n = 385, n (%)	Example of most frequent drug, (n)
Cardiovascular system	106 (27.5)	Atorvastatin (26), simvastatin (10), amlodipine (10), metoprolol (9), enalapril (9)
Anti-infectives for systemic use	104 (27.0)	Piperacillin and tazobactam (11), ceftazidime (10), azithromycin (10), meropenem (7), levofloxacin (6)
Alimentary tract and metabolism	61 (15.8)	Omeprazole (25), metformin (9), sodium carbonate (4), human Insulin/ Isophane Insulin (3), glipizide (2), calcitriol (2)
Blood and blood forming organs	43 (11.2)	Warfarin (17), folic (6), aspirin (5), clopidogrel (4), enoxaparin (3), ferrous fumarate (4)
Respiratory system	25 (6.5)	Salmeterol and fluticasone (10), formoterol and budesonide (6), tiotropium bromide (3), fenoterol and ipratropium bromide (3), theophylline (3)
Nervous system	19 (4.9)	Phenytoin (4), tramadol (2), nortriptyline (2), lorazepam (1), sertraline (1), fluoxetine (1)
Genitourinary system and reproductive hormones	6 (1.6)	Alfuzosin (4), finasteride (1), tamsulosin (1)
Musculoskeletal system	6 (1.6)	Allopurinol (2), colchicine (2), tolperisone (1), febuxostat (1)
Antiparasitic products, insecticides and repellents	5 (1.3)	Metronidazole (3), hydroxychloroquine (1), ivermectin (1)
Systemic hormonal preparations	4 (1.0)	Prednisolone (3), methimazole (1)
Various	4 (1.0)	Calcium folinate (3), calcium polystyrene sulfonate (1)
Antineoplastic and immunomodulating agents	2 (0.5)	Leflunomide (1), methotrexate (1)

*ATC* anatomical therapeutic chemical classification system.

### Pharmacists’ interventions to resolve the DRPs

In total, pharmacists directly provided 309 interventions in order to solve the DRPs (Table 4). Interventions occurred mainly at both prescriber and drug levels. At prescriber level, the most frequent intervention was intervention proposed to prescribers (n = 203, 65.7%) followed by discussion with prescribers (n = 90, 29.1%). The physicians were informed or asked for additional information (n = 16, 5.2%). The major intervention at the drug level was starting a drug (n = 105, 34.0%) for a new condition and/or pre-existing conditions of the patients, as well as re-initiation of the drugs which were discontinued during the acute state. Dosage change found in 90 patients (29.1%) due to patient’s renal function or laboratory results. The discontinuation of drugs as a result of drug duplication or no longer indication accounted for 22.7% of the interventions. Overall, 95.8% (n = 296) of the interventions were accepted from the physician.

**Table 4:** Interventions to resolve the DRPs provided by clinical pharmacists.

Domain	Intervention	Total, n = 309, n (%)
At prescriber level	Prescriber informed only	14 (4.5)
	Prescriber asked for information	2 (0.7)
	Intervention proposed to prescriber	203 (65.7)
	Intervention discussed with prescriber	90 (29.1)
At drug level	Drug changed to	14 (4.5)
	Dosage changed to	90 (29.1)
	Formulation changed to	8 (2.6)
	Instructions for use changed to	12 (3.9)
	Drug discontinued	70 (22.7)
	Drug started	105 (34.0)
	Monitoring	10 (3.2)

## Discussion

This is the first study that analyzed and compared DRP in different stages of hospital stay in hospitalized patients at a tertiary hospital in Thailand. A total of 257 patients had at least one DRP with median of 1 (range 1–3) DRPs per patient. 46.8% of the total 316 DRPs were likely to occur at discharge than at other stages. No drug treatment in spite of existing indication and dose too high were the most frequent subcategories leading to DRPs. No drug treatment despite existing indication was detected at discharge approximately 56.1% compared with the remaining two stages (14.3% at admission and 8.8% during hospital stay). The most common intervention was starting a drug. The physician’s acceptance rate was 95.8%. These finding demonstrate the need for tailored pharmaceutical care services in hospitalized patients to optimize drug use.

The incidence of DRPs reported in this study is consistent with the report of previous studies, with DRPs incidence ranging from 15.5 to 81.0% in hospitalized patients^6,7,9–12^. Such variation may be described by different clinical contexts as well as definitions and methods used to identify the DRPs. A study by Garin et al. reported proportions of patients with DRPs around 45.1% among inpatients admitted to the medical wards (such as internal medicine, geriatrics, neurology, gastroenterology, and pulmonology)^7^, while the present study exclusively focused on the patients in internal medical wards. The usual care and drug prescribing pattern may differ between settings. Additionally, the PCNE classification used in this study is different from Strand et al. classification system used in other studies^6,9^.

In this study, the most frequent problems leading to DRP were untreated symptoms/indication and adverse drug events. Similar results have been shown in previous studies^7,9^. The untreated symptoms/indication occurred when patients did not receive the drugs recommended in standard treatment for their conditions. It was consistent with our study, in which no drug treatment in spite of existing indications was the predominant cause. DRPs were mostly caused by failure to restart the drugs for patients having underlying disease. These were taken place after drug discontinuation during the acute phase of illness, such as antidiabetic drugs, antihypertensive drugs. In addition, some patients did not receive the drugs, being at risk of developing a new condition. For example, the use of proton-pump inhibitors as a prophylaxis for gastrointestinal bleeding in a patient treated with antithrombotic therapy or prolonged use of ventilator. Dose too high was accounted for 22.8% of the total DRPs. The majority of these cases were international normalized ratio (INR) higher than therapeutic level because of inappropriate dosing of warfarin and failure to adjust the dose of drugs such as piperacillin/tazobactam, ceftazidime and meropenem based on patient’s renal function. Based on the fact that decline of renal function can result in inappropriate drug use^12,23^.

The drug classes that often causing a DRP were cardiovascular system, anti-infective for systematic use, and alimentary tract and metabolism. Similarly, a study conducted at medical wards of southwestern Ethiopian hospitals found that ceftriaxone was the most frequent drug for DRPs^24^. The study conducted in university hospitals in Spain showed that the five most frequent drug classes in the detected DRPs were anti-infectives for systemic use, nervous system, cardiovascular system, blood and blood forming organs, and alimentary tract and metabolism^7^. In contrast, a prospective study conducted in general hospitals in Norway showed that the most common drugs associated with DRPs were warfarin, digoxin, and prednisolone^6^. The wide variety of drugs involved in DRPs was due to drug prescribing patterns among individual physicians and different treatment guidelines.

Considering the stage of hospital stay related to DRPs**,** our study found that DRPs can be detected at any stages of the hospital stay; however, most of these DRPs were likely to occur at hospital discharge than at other stages. While the study of Geeson et al. found that clinically relevant MRPs occurred more frequently at admission^19^. However, it is difficult to directly compare these results due to differences in methodology of study. Clinical pharmacist working at study sites may have a role to play at different DRPs among different stages of the hospital, such as focusing on discharge screening or newly admitted patients.

We found that each stage showed an individual DRP pattern. This is based on the fact that each stage of hospital stay may be related to prescribing drugs. The subcategory of no drug treatment in spite of existing indication was found at high percentage of DRPs at discharge, which is consistent with previous study that omission of drug was the common medication error on hospital discharge prescription^25,26^. Our study also found that no drug treatment despite existing indication around 16.7% at admission. Therefore, pharmaceutical care service such as medication reconciliation is very useful at hospital admission and discharge^25,27^. While drug safety checks including recommendations for dose adjustment (e.g., antibiotics and warfarin) and inappropriate drug use should be included in PCS during the hospital stay. All these differences highlight the need to tailor the PCS of a clinical pharmacist working on the hospital ward in each stage.

There are similarities between our findings and previous studies such as the most common interventions included starting a drug and changing dosage^7,11,28^. The acceptance rate of the pharmacist interventions by physicians in our study was high (95.8%), which is in line with the findings from other studies where the proportion of accepted intervention was more than 80%^9–12,28^. This may reflect clinically relevant suggestions by pharmacists, and the long-standing relationship and trust between pharmacists and physicians. It indicates the role of pharmacists in healthcare team at hospital setting. The clinical pharmacy services in hospitalized patients are needed to enhance drug efficacy and safety.

Strengths of this study include prospective data collection and inclusion of consecutive admissions, this enabled optimal measurement of DRPs. Additionally, this study is the comparative analysis of DRPs with distinct stages of hospital stay. Previous studies focused on DRPs only at admission, transitional care, or at hospital discharge. However, a possible limitation was that this study was conducted at one site only. The results should therefore be generalized with caution. In other hospitals or different patient populations there may be additional factors influencing the characteristics of DRPs.

## Conclusion

Drug-related problems in hospitalized patients can occur at any stage of the hospital stay. Identification and resolution of DRPs in this study were high at hospital discharge compared with other stages of hospital stay. DRP category differed among stages of hospital stay. Our findings provide insight into the type of pharmaceutical care service required. The need for medication review is useful at admission and at discharge to minimize DRPs Strategies to address problems related to dose adjustment are required during hospital stay, together with ongoing clinical pharmacist review.

## Supplementary Information

Supplementary Information 1.

Supplementary Information 2.

## Data Availability

The data generated and/or analyzed during the current study are not publicly available due to ethical concerns and the hospital regulations, but are available from the first author on reasonable request.
