# Supplementary material for: Characteristics of drug-related problems and pharmacist’s interventions in hospitalized patients in Thailand: a prospective observational study
Source: Sci Rep. 2022 Oct 12;12:17107. doi: 10.1038/s41598-022-21515-7 (PMC9556629; doi:10.1038/s41598-022-21515-7)
Supplement: Supplementary file 1 — Supplementary Information 1. [file 41598_2022_21515_MOESM1_ESM.pdf]

Supplementary information 1 Test of normality

			Statistic	Std. Error
Age	Mean		67.30	.979
	95% Confidence Interval for Mean	Lower Bound	65.38	
		Upper Bound	69.23	
	Std. Deviation		15.688	
	Skewness		-.879	.152
	Kurtosis		.813	.303
numco	Mean		2.68	.094
	95% Confidence Interval for Mean	Lower Bound	2.50	
		Upper Bound	2.87	
	Std. Deviation		1.500	
	Skewness		.025	.152
	Kurtosis		-.788	.303
numpremed	Mean		5.72	.234
	95% Confidence Interval for Mean	Lower Bound	5.26	
		Upper Bound	6.19	
	Std. Deviation		3.757	
	Skewness		.385	.152
	Kurtosis		-.084	.303

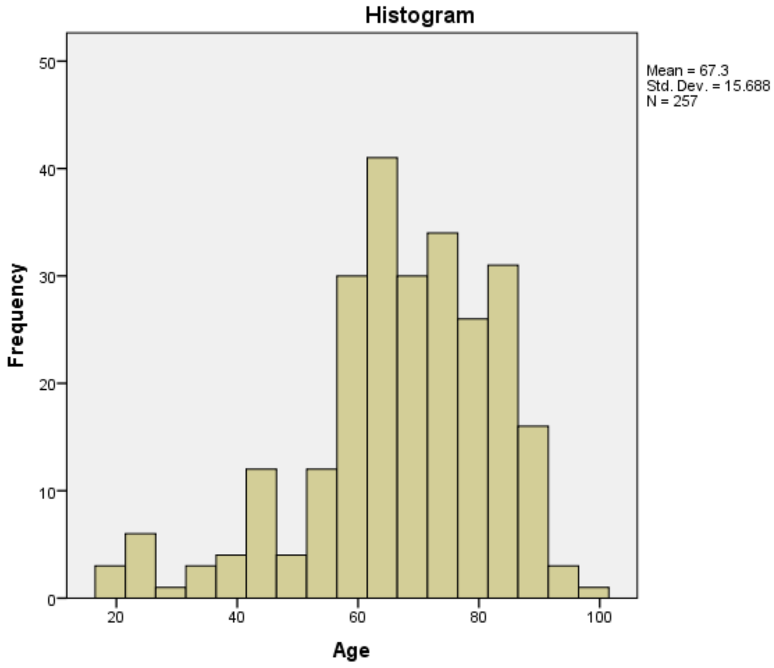

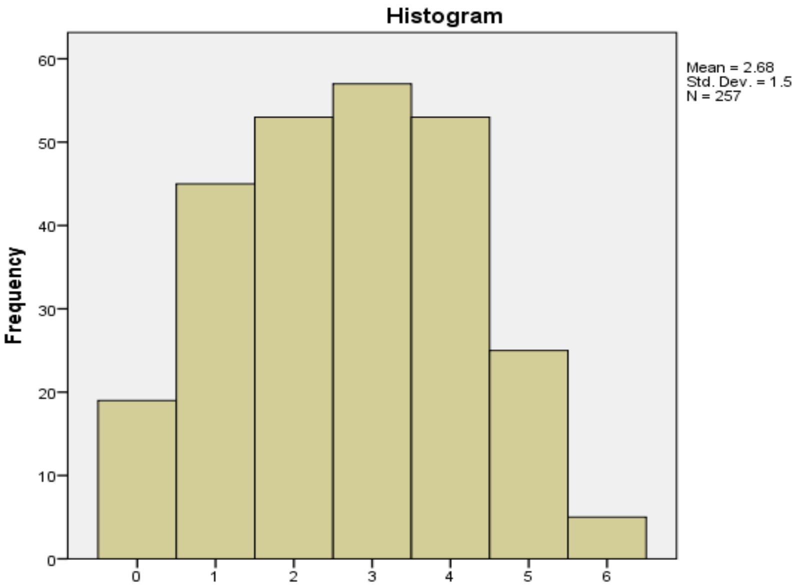

Number of comorbidities

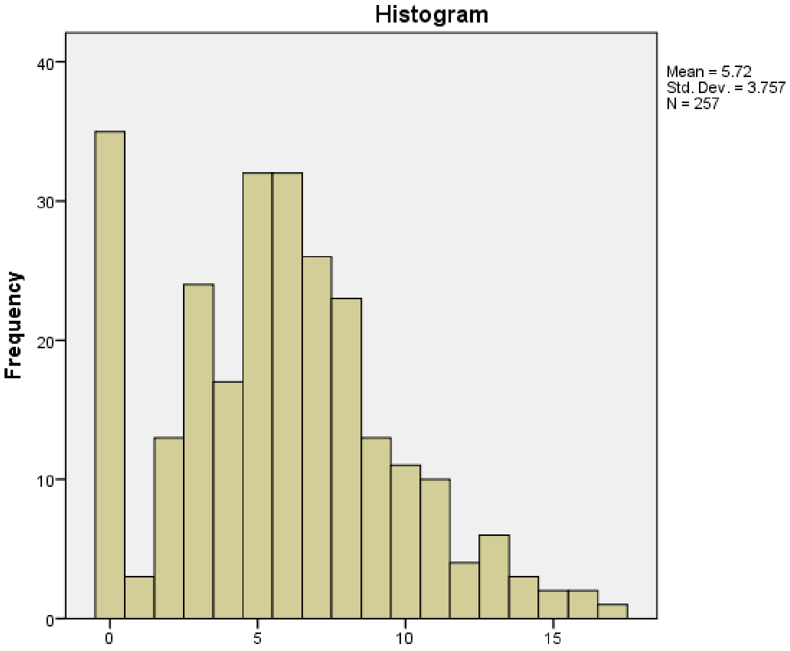

Number of medications used
