# Supplementary material for: Characteristics of drug-related problems and pharmacist’s interventions in hospitalized patients in Thailand: a prospective observational study
Source: Sci Rep. 2022 Oct 12;12:17107. doi: 10.1038/s41598-022-21515-7 (PMC9556629; doi:10.1038/s41598-022-21515-7)
Supplement: Supplementary file 2 — Supplementary Information 2. [file 41598_2022_21515_MOESM2_ESM.pdf]

Supplementary Information 2 Comparison of 95% confidence interval of individual proportion regarding to the stages of hospital stay.

2.1.1 Problems of drug-related problems: using Chi-square test

Category/subcategory of DRP problems according to PCNE v 8.02 classification	95% Confidence interval			p-value (Test for difference among stages)
	At admission n = 77	During the hospital stay n = 91	At discharge n = 148	
**Treatment effectiveness**				
Effect of drug treatment not optimal	0.190, 0.321	0.057, 0.120	0.079, 0.148	0.001[a]
Untreated symptoms or indication	0.060, 0.128	0.051, 0.109	0.388, 0.537	< 0.001[a]
**Treatment safety**				
Adverse drug event (possibly) occurring	0.341, 0.500	0.324, 0.478	0.142, 0.242	< 0.001[a]
**Other problems**				
Unnecessary drug-treatment	0.031, 0.077	0.181, 0.304	0.034, 0.074	< 0.001[a]

DRP drug-related problem, *PCNE* Pharmaceutical Care Network Europe

[a] p-value of Chi-square for comparing three groups, statistical significance 0.05

2.1.2 Problems of drug-related problems: using Fisher's exact test

Category/subcategory of DRP problems according to PCNE v 8.02 classification	95% Confidence interval		p-value (Test for difference among stages)
	During admission (at admission and during the hospital stay) n = 168	At discharge n = 148	
**Treatment effectiveness**			
No effect of drug treatment	0.008, 0.024	0.009, 0.027	1.000[b]

[b] p-value of Fisher's exact test for comparing two groups: one group was DRPs occurred during admission (combination of at admission and during the hospital stay) and another was the DRPs at discharge, statistical significance 0.05

2.2.1 Causes of drug-related problems: using Chi-square test

Category/subcategory of DRP causes according to PCNE v 8.02 classification	95% Confidence interval			p-value (Test for difference among stages)
	At admission	During the hospital stay	At discharge	
**Drug selection**				
Inappropriate drug (within guidelines but otherwise contraindication)	0.060, 0.128	0.032, 0.076	0.012, 0.034	0.026[a]
Inappropriate duplication of therapeutic group or active ingredient	0.017, 0.051	0.097, 0.185	0.016, 0.040	< 0.001[a]
No drug treatment in spite of existing indication	0.076, 0.154	0.044, 0.098	0.415, 0.564	< 0.001[a]
**Dose selection**				
Dose too low	0.092, 0.179	0.026, 0.065	0.027, 0.060	0.006[a]
Dose too high	0.171, 0.295	0.189, 0.315	0.083, 0.154	0.007[a]

DRP drug-related problem, *PCNE* Pharmaceutical Care Network Europe
[a] p-value of Chi-square for comparing three groups, statistical significance 0.05

2.2.2 Causes of drug-related problems: using Fisher's exact test

Category/subcategory of DRP problems according to PCNE v 8.02 classification	95% Confidence interval		p-value (Test for difference among stages)
	During admission (at admission and during the hospital stay) n = 168	At discharge n = 148	
**Drug selection**			
Inappropriate drug according to guidelines/formulary	0.037, 0.077	0.016, 0.040	0.211[b]
No indication	0.008, 0.024	0.009, 0.027	1.000[b]
Inappropriate combination of drugs or drugs and herbal medication	0.002, 0.012	0.003, 0.013	1.000[b]
Too many drugs prescribed for indication	0.023, 0.053	0.006, 0.020	0.111[b]
**Drug form**			
Inappropriate dosage form	0.014, 0.036	0.012, 0.034	1.000[b]
**Dose selection**			
Dosage regimen not frequent enough	0.005, 0.018	0, 0.007	0.500[b]
Dose timing instructions wrong, unclear or missing	0.030, 0.065	0.019, 0.047	0.609[b]

2.2.2 Causes of drug-related problems: using Fisher's exact test (cont.)

Category/subcategory of DRP problems according to PCNE v 8.02 classification	95% Confidence interval		p-value (Test for difference among stages)
	During admission (at admission and during the hospital stay) n = 168	At discharge n = 148	
**Treatment duration**			
Duration of treatment too short	0, 0.006	0.006, 0.020	0.219[b]
Duration of treatment too long	0.011, 0.030	0, 0.007	0.126[b]
**Others**			
Inappropriate outcome monitoring	0.002, 0.012	0, 0.007	1.000[b]
Drug order incorrect or incomplete	0.002, 0.012	0.009, 0.027	0.344[b]

[b] p-value of Fisher exact test for comparing two groups: one group was DRPs occurred during admission (combination of at admission and during the hospital stay) and another was the DRPs at discharge, statistical significance 0.05
